# *Parametarhizium hingganense*, a Novel Ectomycorrhizal Fungal Species, Promotes the Growth of Mung Beans and Enhances Resistance to Disease Induced by *Rhizoctonia solani*

**DOI:** 10.3390/jof8090934

**Published:** 2022-09-02

**Authors:** Ying Gao, Siyu Gao, Yang Bai, Wei Meng, Lijian Xu

**Affiliations:** 1 College of Life Science, Northeast Forestry University, Harbin 150040, China; 2 College of Advanced Agriculture and Ecological Environment, Heilongjiang University, Harbin 150080, China; 3 Key Laboratory of Saline-alkali Vegetation Ecology Restoration (Northeast Forestry University), Ministry of Education, Harbin 150040, China

**Keywords:** *Parametarhizium hingganense*, growth promotion, biocontrol, *Rhizoctonia solani*, P solubilization, mycorrhizae

## Abstract

The mutualistic interactions between mycorrhizae and plants first occurred along with the terrestrialization of plants. The majority of vascular plants are in symbiosis with mycorrhizal fungi. Due to their importance to the economy and ecology, arbuscular mycorrhizal (AM) and ectomycorrhizal (ECM) fungi emerge as the most popular ones. However, the mechanism underlying the beneficial function of ECM fungi is not as clear as AM fungi. Here, the interaction between *Parametarhizium hingganense*, a novel fungal species isolated from forest litter, and mung bean (*Vigna radiata*) was studied. *P. hingganense* demonstrated P solubilization ability in vitro. Treatment of *P. hingganense* on the seeds resulted in promoted growth with enhanced P content. The hyphae of green fluorescence protein (GFP)-tagged *P. hingganense* were found to surround the roots and develop between cells, suggesting the establishment of an ectomycorrhizal symbiosis. Upon symbiosis with *P. hingganense*, the levels of jasmonic acid (JA) and gibberellin (GA_1_) and total phenolic and flavonoid content elevated. Meanwhile, damping off caused by *Rhizoctonia solani* in mycorrhizal plants was alleviated. Taken together, the above findings suggested that symbiosis with *P. hingganense* conferred growth promotion and priming of defense responses to host plants which should be associated with facilitated P uptake and increased JA and GA_1_ levels.

## 1. Introduction

Mutualistic symbiosis with mycorrhizal fungi occurred along with plant terrestrialization [1]. There are four major types of mycorrhizal association: arbuscular mycorrhizal (AM), ectomycorrhizal (ECM), ericoid mycorrhizal, and orchid mycorrhizal [2]. Most of the vascular plants are in symbioses with AM fungi (72%), and only 2%, 1.5%, and 10% are associated with ECM, ericoid mycorrhizal, and orchid mycorrhizal fungi, respectively [3]. Amongst mycorrhiza-forming fungi, AM and ECM are intensively studied. The hyphae of both AM and ECM fungi colonize the surface of the root [2]. However, inside the plant tissues, AM fungal hyphae penetrate into the living cells and form highly branched arbuscules, while ECM fungal hyphae develop only between epidermal cells [2]. Fossil records reveal that associations of AM fungi with plants are ancient events that can be traced back to at least 450 million years ago with a single origin, while ECM fungi associations occurred in the Cretaceous and evolved independently with multiple origins [3].

Growing evidence shows that mycorrhizal symbiosis improves the performance of host plants, mainly through growth promotion and elevated plant resistance to biotic and abiotic stresses [2,4,5]. First, both AM and ECM fungi can deliver nutrients to the host plants, particularly P which is one of the least mobile plant macronutrients in soil [6,7]. Generally, the availability of P in soil is extremely low [6]. In AM fungi, high-affinity inorganic phosphate (Pi) transporters locate on the extraradical hyphae and in the colonized cortical cells, which function in the uptake of Pi from soil and translocation into the host plants [2,5]. In contrast, only a few Pi transporters are characterized in ECM fungi [8]. However, ECM fungi influence Pi uptake through enzyme production and organic acid excretion, facilitating the utilization of insoluble Pi and organic phosphate, which are not preferred by AM fungi [6,7]. Second, upon mycorrhizal symbiosis, endogenous levels of phytohormones are affected, including growth hormones (auxin and gibberellins (GAs)) and defense hormones (jasmonic acid (JA), salicylic acid (SA), abscisic acid (ABA)). Auxin appears to function throughout the lifespan of plants, from embryogenesis to senescence [9]. GAs are essential to plant growth and development, including seed germination, stem elongation, leaf expansion, flower initiation, and flower and fruit development [10]. Furthermore, sufficient GA signaling positively regulates AM fungal hyphal branching and facilitates AM fungi colonization on the host plant [11]. DELLA proteins play central roles during AM symbiosis development and in the cross-talk among GA, ABA, and Indole-3-acetic acid (IAA) signaling pathways [12]. Despite the function of SA and JA signaling in mycorrhiza development being ambiguous, available evidence shows that both AM and ECM fungi primed defense responses to stresses are associated with the activation of SA and JA signaling pathways, although most of the findings are generated from AM symbiosis [12,13,14,15]. In contrast to the diverse plant species (most of them are crops) and fungal strains involved in AM symbiosis, limited plant species appear in research into ECM–plant interaction, and the interaction between poplar and *Laccaria bicolor* is extensively investigated [4]. However, unraveling the mechanism underlying the ECM-induced defense responses is progressing [4].

*Rhizoctonia solani* is a soil-borne phytopathogen with a wide range of hosts and worldwide distribution. It can cause sheath blight on rice [16], seed rot and root lesions on radish [17], black scurf and stem canker on potato [18], damping off on cotton [17], root rot and damping off on mung bean [19,20], etc. The *R. solani* complex can be further divided into anastomosis groups (AGs), and 13 AGs have been recognized [18,21]. For example, AG-1 can cause seed and hypocotyl rot, as well as damping off of many plant species such as rice and mung bean [16,17]. It has been reported that *R. solani* can cause a serious disease on mung bean and yield loss up to 57% [20].

*Parametarhizium*, a fungal genus in the Clavicipitaceae family which was originally isolated from forest litter and erected in 2021, includes *P. hingganense* and *P. changbaiense*, both of which possess anti-insect activities [22]. Fungi in Clavicipitaceae show diverse lifestyles including soil saprotrophs, plant pathogens, plant symbionts, and invertebrate parasites such as *Epichloë* spp. (symbionts of grasses), ergot fungi (*Claviceps* spp.) that parasitize ears of cereals, and *Metarhizium* spp. (parasites of insects and plant symbionts) [23,24,25]. Recently, it was found that *Metarhizum* spp. could be plant symbionts to promote plant growth and enhance plant resistance to disease [26]. *Parametarhizium* as a newfound genus from forest litter is phylogenetically close to the genus *Metarhizium*. Consistent with *Metarhizum*, *Parametarhizium* spp. are also entomopathogenic fungi [22]. However, it remains unknown whether *Parametarhizium* spp. are plant symbionts and what the influences of *Parametarhizium* spp. on the performance of plants are during the plant–*Parametarhizium* spp. interactions.

Here, we demonstrated that *P. hingganense* established an ectomycorrhizal symbiosis with mung bean roots. *P. hingganense* colonization significantly promoted the growth of mung beans with elevated Pi content and altered phytohormone levels. Furthermore, mycorrhizal symbiosis conferred resistance to disease caused by *R. solani*. Collectively, our findings provide evidence of the influence of a new fungal species from forest litter, *P. hingganense*, on host crop plants and an alternative biocontrol agent to promote plant growth and enhance plant disease resistance. 

## 2. Materials and Methods

### 2.1. Fungal and Plant Materials

*P. hingganense* (CGMCC 19143) and *R. solani* AG-1 (CCTCC AF 2021031) were maintained on Potato Dextrose Agar (PDA) medium at room temperature. Isolation and identification of *P. hingganense* were described in detail by [22]. To make the conidial suspension, *P. hingganense* was cultured under a 16-h light/8-h dark cycle for 10 days. The conidia were eluted with sterile 0.05% Tween-20 solution and filtered through four layers of gauze. The final conidia concentration was adjusted to 1 × 10^8^ conidia mL^−1^ with ddH_2_O. Seeds of mung bean (*Vigna radiata*) variety Lvfeng-2 were purchased from the local supermarket and are susceptible to *R. solani*.

### 2.2. Experimental Design

To examine the effect of *P. hingganense* treatment on the growth of mung beans, seeds were surface sterilized with 75% ethanol for 1 min and rinsed with sterilized ddH_2_O five times. Seeds were subsequently placed into a 9-cm Petri dish filled with *P. hingganense* conidial suspension which was controlled to cover the top surface of the seeds. Seeds were soaked in the dark for 24 h, followed by 2–3 days growth in the conidial suspension under a 16-h light/8-h dark cycle at 25 °C (until the length of the radicle was 1.5 cm). The loss of conidial suspension due to imbibition and evaporation was supplemented by sterilized ddH_2_O. The 0.05% Tween-20 solution was used as a control. Germinated seeds with a similar growth stage were transferred to the pots filled with sterilized soil and vermiculite mixture (potted soil containing 25.05 mg kg^−1^ nitrate nitrogen, 0.69 mg kg^−1^ ammonium nitrogen, 1.18 mg kg^−1^ available phosphorus, 292.25 mg kg^−1^ available potassium, total nitrogen 10.2 mg g^−1^, total phosphorus 8.59 mg g^−1^, total potassium 20 mg g^−1^, and organic matter 0.35 g g^−1^, pH = 6.96 [27], was mixed with vermiculite (1:1) and sterilized at 121 °C for 40 min). One pot contained one germinated seed which grew under a 16-h light/8-h dark cycle at 25 °C. In order to observe the growth rate, the heights of 20 seedlings each from the *P. hingganense*-treated and control group were measured with a ruler daily using the top edge of the pot as a reference. Meanwhile, root area, leaf area, root length, stem length, and total biomass of 21-day-old plants from the 25 pots of both the *P. hingganense*-treated group and the control group were measured or analyzed using Image J software (ImageJ 1.53a, Wayne Rasband, National Institutes of Heath, Bethesda, MD, USA, http://imagej.nih.gov/ij, accessed on 7 August 2022) [28]. Further experiments included observation of the colonization of *P. hingganense* on the roots and the influence of *P. hingganense* treatment on the levels of chlorophyll, Pi, and hormones in plants.

To investigate the function of *P. hingganense* treatment on disease resistance, four treatments were applied on mung bean plants (4–5 cm in height): (i) control plants with no *P. hingganense* treatment and *R. solani* inoculation; (ii) *P. hingganense*-treated plants with no *R. solani* inoculation; (iii) control plants inoculated with *R. solani* without previous *P. hingganense* treatment; and (iv) *P. hingganense*-treated plants with *R. solani* inoculation. For *R. solani* inoculation, a 0.5-cm fungal disc was placed in the middle of a 9-cm PDA plate and cultured for 2 days at room temperature. Subsequently, 0.5-cm fungal discs from the actively growing edge of the fungal culture were prepared. Using forceps to make a hole in the soil 1 cm in depth and 2 cm from the plants, a 0.5-cm fungal disc was then inoculated into the hole with the fungal culture side facing the plant. All plants continued to grow under a 16-h light/8-h dark cycle at 25 °C. Further experiments included analysis focusing on the growth comparison of 20 pots of plants from the four respective treatments, the biocontrol effect of *P. hingganense*, and differences in the content of malondialdehyde (MDA) and total phenolic and flavonoid content. 

Except for the specified number of plants used for analysis, all measurements were conducted with three replicates per treatment, each replicate containing at least 10 plants.

### 2.3. Phosphate Solubilization

The potential of *P. sola* in relation to phosphate solubilization was measured by its ability to solubilize insoluble inorganic phosphate tricalcium phosphate (TCP, Ca_3_(PO_4_)_2_). According to [29], a 0.5-cm fungal disc from the edge of a fungal colony was placed in the middle of 6-cm Petri dish containing the National Botanical Research Institute’s phosphate growth medium (NBRIP) [30] (10 g glucose, 5 g Ca_3_(PO_4_)_2_, 5 g MgCl_2_·6H_2_O, 0.25 g MgSO_4_·7H_2_O, 0.2 g KCl, 0.1 g (NH_4_)_2_SO_4_, 20 g agar, and 1000 mL distilled water) and was cultured for 14 days at room temperature. A PDA disc was used as a control. The phosphate solubilization index was calculated from the values of three replicates using the following formula:

Solubilization index = (colony diameter + clearing zone diameter)/colony diameter(1)

### 2.4. Pi Content Assay

The Pi content in the plants was determined according to [31]. Briefly, roots and aerial parts (stems and leaves) of 21-day-old plants were freshly collected and weighed. Samples were ground with liquid nitrogen and homogenized at the ratio of 1 mg of fresh sample to 10 μL of extraction buffer (10 mM Tris, 1 mM EDTA, 100 mM NaCl, 1 mM β-mercaptoethanol, and 1 mM phenylmethylsulfonyl fluoride, pH 8.0). A total 900 μL of 1% glacial acetic acid was added to 100 μL of homogenized sample and incubated at 42 °C for 30 min. After centrifugation at 13,000× *g* for 5 min, the supernatant was harvested. For the Pi assay, 300 μL of the supernatant was mixed with 700 μL of assay solution (0.35% NH_4_MoO_4_, 0.86 N H_2_SO_4_, and 1.4% ascorbic acid) and incubated at 42 °C for 30 min. The Pi content was determined by A_820_.

### 2.5. Quantification of IAA in Fungal Culture

A fungal disc (0.5 cm) of *P. hingganense* was inoculated into the PD liquid medium supplemented with 0.1% (*w*/*v*) L-tryptophan and shaken at 180 rpm for 20 days at room temperature. PD medium without L-tryptophan was used as a control. The fungal culture was sampled at 5, 10, 15, and 20 dpi. IAA content in the fungal culture was examined according to [32]. The fermented broth was centrifuged at 12,000× *g* for 15 min. Subsequently, 1 mL of supernatant was mixed with 0.2 mL of phosphoric acid and 2 mL of Salkowski reagent (50 mL 35% HClO_4_, 1 mL 0.5 M FeCl_3_) and incubated for 20 min in the dark at room temperature. The IAA content was calculated using the absorbance at 535 nm with a standard IAA curve (10–100 μg mL^−1^).

### 2.6. Construction of Green Fluorescence Protein (GFP)-Tagged P. hingganense

Using pBI-eGFP as a template, a 0.73-kb GFP PCR fragment was obtained using primer pairs 1307GFP_F: 5′-AAGCTTATCGATACCGTCGACATGGTGAGCAAGGGCGAGGAG-3′ and 1307GFP_R: 5′-TTTGCGGAGTACCCGGGTACCTTACTCGAGCTTGTACAGCTC-3′. The GFP fragment was subsequently ligated into the *Sal* I and *Kpn* I site of pCAMBIA1307 using an In-Fusion HD kit (Clontech, Mountain View, CA, USA) to generate the pCAMBIA1307-eGFP plasmid, which was subsequently transferred to *Agrobacterium tumefaciens* GV3101. GFP-tagged *P. hingganense* was accomplished through *A. tumefaciens*-mediated transformation [33]. Briefly, a single colony of GV3101 harboring the pCAMBIA1307-eGFP plasmid was inoculated into 2 mL LB liquid medium (supplemented with 50 µg mL^−1^ kanamycin and 50 µg mL^−1^ gentamicin) and was shaken at 200 rpm for 16–20 h at 28 °C. Cells were harvested by centrifugation at 12,000 rpm for 2 min and washed twice with IMAS broth. The cell culture was adjusted to an OD660 nm of 0.15, followed by incubation to an OD660 nm between 0.6 and 0.8. The conidial suspension of *P. hingganense* was collected by scraping fungal culture with 5 mL of sterile 0.05% Tween-20. After centrifugation at 12,000 rpm for 2 min, conidia were washed twice with sterile water and adjusted to 1 × 10^6^ conidia mL^−1^. The mixture of 500 μL conidial suspension and 500 μL Agrobacterium was incubated for 1 h at 28 °C with shaking (180 rpm). A 30 μL aliquot of the mixture was spread on IMAS agar and co-cultivated at 28 °C for 2–3 days. The culture was subsequently covered with M-100 (supplemented with 300 μg mL^−1^ cefotaxime and 100 μg mL^−1^ hygromycin B) and incubated at 25 °C for 2–3 weeks. The positive colonies were screened on PDA supplemented with 100 μg mL^−1^ hygromycin B and 300 μg mL^−1^ cefotaxime and examined under a fluorescence microscope. 

### 2.7. Observation of the Colonization

To observe the symbiosis, seeds of mung bean were treated with GFP-tagged *P. hingganense* conidial suspension as described above, then grown hydroponically in Yoshida nutrient solution. Root segments were cut into 2–3 cm sections and fixed in FAA solution (formalin/glacial acetic acid/70% alcohol, 18:1:1, *v*/*v*/*v*) for 12 h at 4 °C. Subsequently, samples were heated to become transparent in 10% KOH at 90 °C and observed in 60% glycerol under a fluorescence microscope. To stain the roots with trypan blue, transparent roots were completely immersed in 0.05% trypan blue solution for 3 min, followed by a rinse with ddH_2_O before being observed under a light microscope.

### 2.8. Quantification of Plant Hormones

Plant hormone extraction and quantification were performed according to [34,35]. Briefly, 21-day-old aerial tissues and roots were harvested and weighed separately. Samples were ground into fine powder in liquid nitrogen and subjected to extraction with 10 mL isopropanol/H_2_O/concentrated HCl (2:1:0.002, *v*/*v*/*v*) supplemented with 8 μL internal standards solution containing 1 μg mL^−1^ of each internal standard compound (d_5_-IAA, d_6_-ABA, d_6_-SA, H_2_JA, and d_2_-GA_4_). After incubation at 4 °C for 30 min with shaking, 20 mL dichloromethane was added to the extraction buffer and incubated for another 30 min at 4 °C with shaking. Samples were centrifuged at 13,000× *g* for 5 min. The lower phase was concentrated to dryness with nitrogen flow and resolved in 400 μL methanol containing 0.1% formic acid. Hormone quantification was completed using an HPLC-MS/MS (6500 QTRAP, AB Sciex, Framingham, MA, USA) equipped with a Poroshell 120 SB-C18 column (Agilent, Santa Clara, CA, USA).

### 2.9. Dual Culture of P. hingganense and R. solani

A dual culture of *P. hingganense* and *R. solani* was formulated according to [36]. A 0.5-cm disc of *P. hingganense* was placed on the left side of a PDA plate and incubated at 25 °C for 6 days. Subsequently, a disc of *R. solani* that was the same size was placed on the right side of the plate. The culture plate was incubated at 25 °C for 7 days. 

### 2.10. Biocontrol Effect of P. hingganense

In accordance with [37,38], on the 7th day after inoculation with *R. solani*, the wilting index, the hypocotyl lesion index, and the plant lodging index were used to measure the biocontrol effect of *P. hingganense*. The wilting was rated on a scale of 0 to 4. Grade 0 was a healthy plant and showed no wilting. Grade 1 represents leaves that were slightly wilted. Grade 2 represents leaves that were moderately wilted and drooping. Grade 3 represents leaves that were seriously wilted and drooping and a plant that was obviously inclining. Grade 4 represents a that plant is completely lodging and dying. A hypocotyl lesion was rated on a scale of 0 to 4 according to the hypocotyl lesion area of each plant. Grade 0 represents that no lesion was observed. Grade 1 represents that the lesion area was less than 25%. Grade 2 represents that the lesion area ranged from 25% to 50%. Grade 3 represents that the lesion area ranged from 50% to 75%. Grade 4 represents that the lesion area was more than 75%. Plant lodging was rated on a scale of 0 to 4 according to the inclination angle of the plant to a vertical axis. Grade 0 represents an inclination angle less than 15°. Grade 1 represents an inclination angle that ranged from 15° to 30°. Grade 2 represents an inclination angle that ranged from 30° to 45°. Grade 3 represents an inclination angle that ranged from 45° to 60°. Grade 4 represents an inclination angle that was more than 60°. The wilting index, the hypocotyl lesion index, and the plant lodging index were calculated by the following formula:

Index = ∑(grade × plants of each grade)/(4 × total plants)(2)

Index means the wilting index, the hypocotyl lesion index, or the plant lodging index. Grade means the degree of wilting, hypocotyl lesion, or plant lodging scale, e.g., 0, 1, 2, 3, or 4. 

### 2.11. Measurement of the Chlorophyll, MDA, and Total Phenolic and Flavonoid Content

Total chlorophyll content was measured according to [39] and leaves from *P. hingganense*-treated and non-treated plants were weighed and immersed in 80% ethanol for 24 h in darkness at 25 °C. The absorbance at 663 nm and 645 nm was recorded.

To measure MDA content, aerial tissues and roots were collected and weighed before and 7 days post *R. solani* inoculation, respectively. Samples were ground into fine powder in liquid nitrogen and immediately homogenized with a pre-cooled boric acid buffer solution (pH = 8.8, containing 0.1% β-mercaptoethanol) in a ratio of 1:10. After centrifugation at 13,000 rpm at 4 °C for 20 min, the supernatant was harvested as the crude extraction. MDA content was measured according to [39]. Briefly, the mixture of a 500 μL aliquot of the crude extraction with 1 mL of 20% trichloroacetic acid solution containing 0.5% thiobarbituric acid was incubated for 30 min at 95 °C followed by an immediate and complete cooling on ice. After centrifugation (13,000 rpm, 4 °C, 10 min), the absorbance at 450 nm, 532 nm, and 600 nm was recorded.

To measure total phenolic and flavonoid content, samples were ground into powder in liquid nitrogen and immediately transferred to the pre-cooled 95% methanol solution in a ratio of 1 mL 95% methanol extracts:10 mg fresh weight. The mixture was incubated for 48 h in the dark with continuous shaking at room temperature. After centrifugation at 13,000× *g* for 5 min, the supernatant was collected for measurement. Total phenolic content was quantified according to [40]. A 100 μL aliquot of the supernatant or standard was mixed with 200 μL of 10% (*v*/*v*) Folin–Ciocalteu reagent and vortexed immediately. A total of 800 μL of 700 mM Na_2_CO_3_ was added to the mixture and incubated at room temperature for 2 h. The absorbance at 765 nm was recorded and the concentration was determined by a standard curve with known concentrations of gallic acid. Total flavonoid content was examined according to [40]. Briefly, a 177.5 μL aliquot of the supernatant or standard was mixed with 7.5 μL of 5% NaNO_2_ and incubated for 6 min. After adding 15 μL of 10% AlCl_3_·6H_2_O, the mixture was incubated for 5 min, then 50 μL 1 M NaOH was added. The absorbance at 510 nm was recorded and the concentration was determined by a standard curve with known concentrations of quercetin.

### 2.12. Statistical Analysis

The data obtained here were analyzed using Excel (Office 2019, Microsoft, Redmond, WA, USA). Data were shown as mean ± standard deviation (SD) from three replicates or a specified number of plants. The significant difference between treatments was calculated using Student’s *t*-tests: * *p* < 0.05, ** *p* < 0.01, *** *p* < 0.001.

## 3. Results

### 3.1. P. hingganense Treatment on Seeds Promotes the Growth of Mung Bean Plants

**Figure 1:**
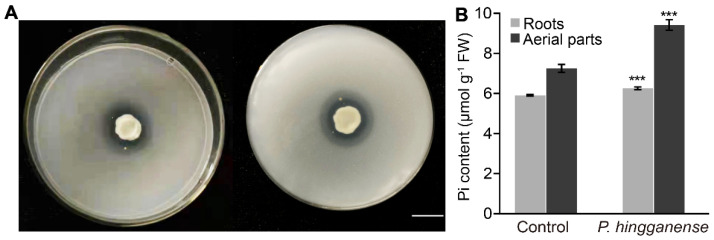
Enhanced inorganic phosphate (Pi) content in *Parametarhizum hingganense*-treated mung bean plants. (**A**) Clear zone of tricalcium phosphate solubilization on the National Botanical Research Institute’s phosphate growth medium (NBRIP) produced by *P. hingganense* (left, front view; right, bottom view). Bar, 1cm. (**B**) Pi content in *P. hingganense*-treated and non-treated mung bean plants. Values are means ± SD (*n* = 3). Asterisks indicate significant differences as evaluated by Student’s *t*-tests: *** *p* < 0.001.

In vitro phosphate solubilization indicated that *P. hingganense* solubilized P on the NBRIP medium with a solubilization index of 2.32 ± 0.18 (Figure 1A), suggesting *P. hingganense* may potentially promote the utilization of insolubilized phosphorus in soil and provide more nutrients (P) for plants to grow. To verify our hypothesis, mung bean seeds were treated with *P. hingganense*. After 21 days of growth in the soil, the Pi content in plants was measured. As expected, Pi content in both the aerial parts and roots of *P. hingganense*-treated plants was significantly higher (+6% in roots; +30% in aerial parts) than non-treated plants (Figure 1B). Meanwhile, Pi levels in the roots were lower than those in aerials parts for both situations (Figure 1B). Together, these findings suggested that *P. hingganense* could solubilize phosphorus and that *P. hingganense* treatment provided more Pi for plants.

**Figure 2:**
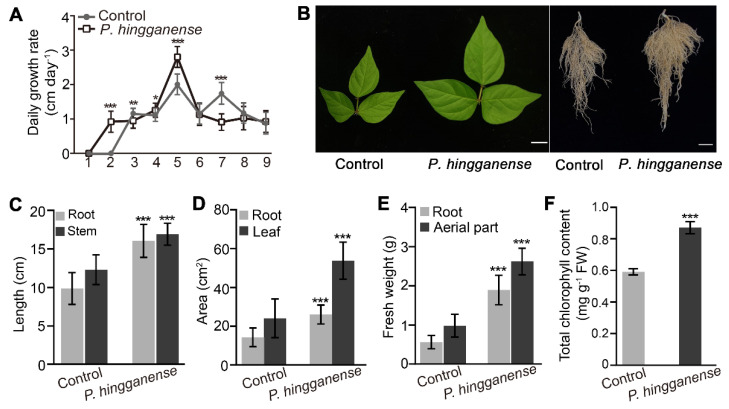
Growth promotion of *P. hingganense* on mung bean plants. (**A**) Comparison of the daily growth rate of *P. hingganense*-treated and non-treated plants within 9 days of planting in soil. Values are means ± SD (n = 20). (**B**–**F**) Phenotypes of *P. hingganense*-treated plants. (**B**) Growth of 21-day-old mung bean plants. Bars, 2 cm. (**C**) Lengths of the root and stem; (**D**) root and leaf area. (**E**) Fresh weight. (**F**) Total chlorophyll content. Values are means ± SD (n = 25). Asterisks indicate significant differences as evaluated by Student’s *t*-tests: * *p* < 0.05, ** *p* < 0.01, *** *p* < 0.001.

Given that P is one of the mineral nutrients essential for plant growth, we next considered whether increased Pi levels due to *P. hingganense* treatment promoted the growth of mung bean plants. Therefore, the growth and phenotype of *P. hingganense*-treated mung bean plants were monitored and compared to non-treated ones. The results obtained showed that significant growth promotion was observed in *P. hingganense*-treated plants (Figure 2). Daily growth rate within 9 days of planting in soil showed that *P. hingganense* treatment accelerated the early growth of mung beans on the 2nd and 5th day, though this was lower on the 7th day and maintained a similar growth rate to the control on the rest of the days (Figure 2A). In comparison to the non-treated plants, the length (+62.6% in roots, +37.3% in stems), area (+81.4% in roots, +123% in leaves), fresh weight (+233.6% in roots, +166.4% in aerial parts), and chlorophyll content (+47.4%) were all increased in *P. hingganense*-treated plants (Figure 2B–F). The above results indicated that the treatment of *P. hingganense* on seeds could promote the growth of mung bean plants. However, whether the increased Pi level was the only factor influencing growth promotion was unclear. 

### 3.2. Symbiotic Relationship between P. hingganense and Mung Bean Plants

**Figure 3:**
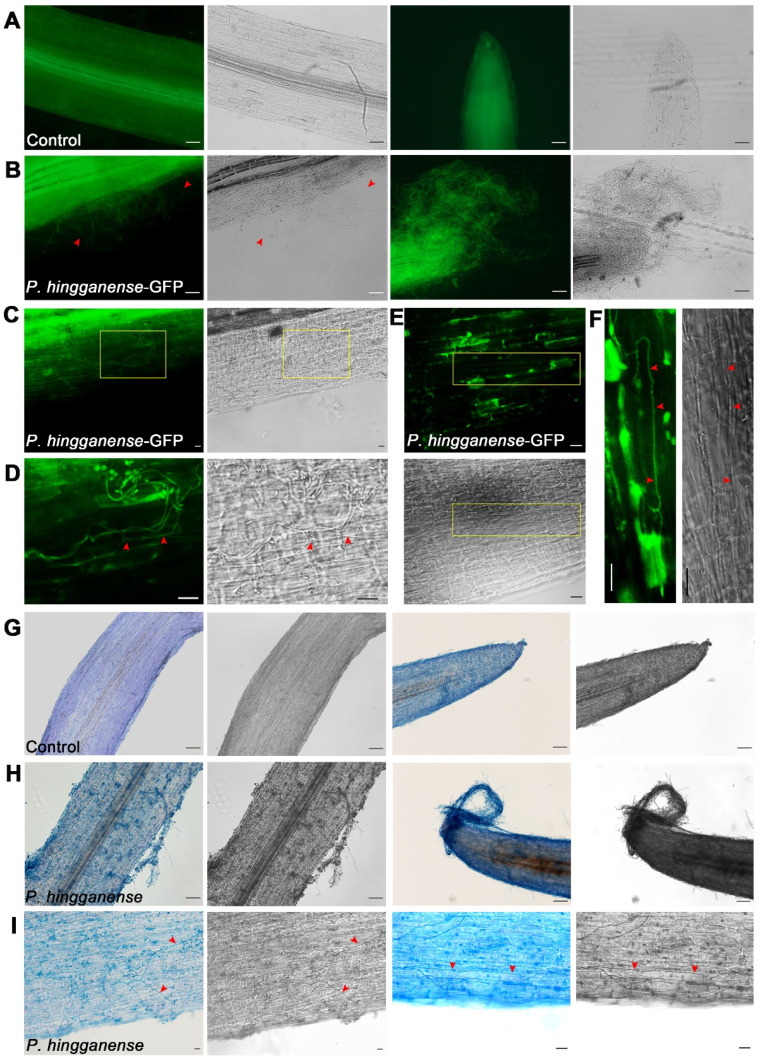
Colonization of *P. hingganense* on the roots of mung bean plants. (**A**–**F**) Fluorescence microscope images showing that the hyphae of green fluorescence protein (GFP)-tagged *P. hingganense* surrounded the roots and developed between the adjacent cells at 19 dpi. (**A**) Root of a non-treated plant. Bars, 100 μm. (**B**) GFP-tagged *P. hingganense*-treated root. Bars, 100 μm. Red arrow heads, hyphae. (**C**) Hyphae developed on the surface of the root. Bars, 20 μm. Yellow rectangles indicate root areas that are enlarged. (**D**) Enlargement of the rectangle in (**C**). Bars, 20 μm. Red arrow heads, hyphae. (**E**) Hypha located between the adjacent cells. Bars, 20 μm. Yellow rectangles indicate root areas that are enlarged. (**F**) Enlargement of the rectangle in (**E**). Bars, 20 μm. (**G**–**I**) Light microscope images of trypan blue staining demonstrating hyphae of *P. hingganense* was colonized on the surface of the root and between the adjacent cells. (**G**) Root of the non-treated plant. Bars, 100 μm. (**H**) *P. hingganense*-treated root. Bars, 100 μm. (**I**) Hyphae on the surface (left) and between the cells (right). Bars, 20 μm. Red arrow heads, hyphae.

To advance our understanding of the impacts of *P. hingganense* on mung bean plants, the location of *P. hingganense* in mung bean plants was visualized by GFP-tagged *P. hingganense*. Detectable GFP fluorescence of the hyphae on the surface of the root was observed at 11 dpi (Figure S1). At 19 dpi, extensive extraradical hyphae enveloped the roots and intercellular hyphae were found between the adjacent cells (Figure 3A–F). Identical colonization of *P. hingganense* on the roots was also displayed by trypan blue staining (Figure 3G–I). The fact that colonization of *P. hingganense* resided within the mung bean plant tissues and on the surface of the roots without apparent disease symptoms, in addition to growth promotion, suggested the potential of *P. hingganense* as a beneficial mycorrhizal fungus.

### 3.3. P. hingganense Colonization Elevates the Content of JA and GA in the Roots

To further elucidate the mechanism of growth promotion by *P. hingganense*, we initially examined the capacity of auxin production by *P. hingganense*, since auxin secreted by fungi is a well-known strategy for facilitating plant growth in plant–fungi symbiosis [41]. Here, the content of IAA was quantified. Nonetheless, IAA was absent in the liquid culture of *P. hingganense* (Figure S2), indicating *P. hingganense* could not produce auxin to boost growth.

**Figure 4:**
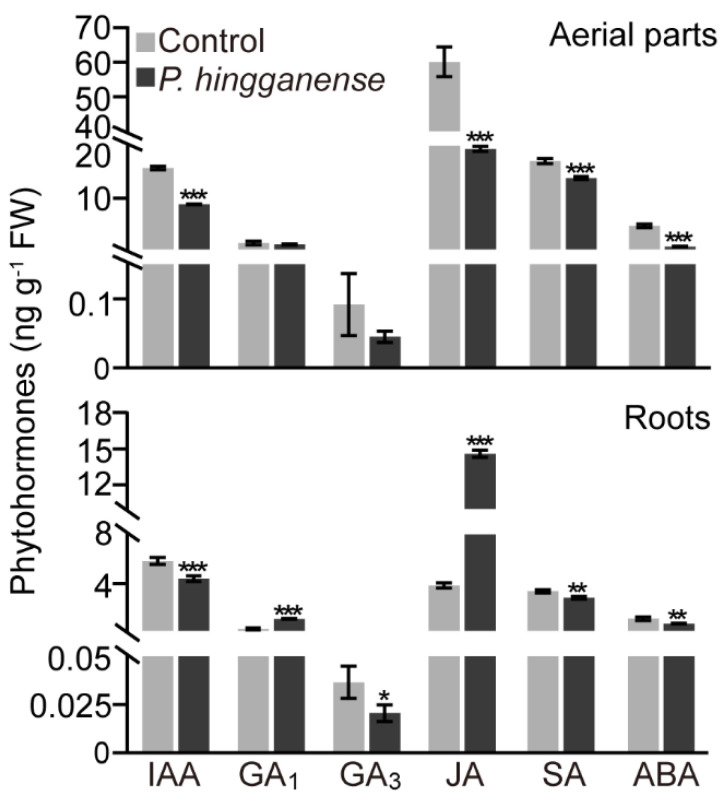
Impacts of *P. hingganense* on phytohormone levels in mung bean plants. Measurement of phytohormones from 21-day-old roots and aerial parts (stems and leaves). Values are means ± SD (n = 3). Asterisks indicate significant differences as evaluated by Student’s *t*-tests: * *p* < 0.05, ** *p* < 0.01, *** *p* < 0.001. IAA, indole-3-acetic acid; GA, gibberellin; JA, jasmonic acid; SA, salicylic acid; ABA, abscisic acid.

Subsequently, the endogenous levels of growth hormones (IAA and GAs) and defense hormones (SA, JA, and ABA) in the roots and aerial parts of mung bean plants were examined. In aerial parts, mycorrhizal colonization decreased the levels of IAA, SA, JA, and ABA, but no significant difference in the levels of GAs was detected between the non-treated and mycorrhizal plants (Figure 4). Meanwhile, the influence of the *P. hingganense* colonization on the levels of different hormones in aerial parts varied. The content of JA was affected seriously and was about 3-fold lower than non-treated plants. In the roots, the level of IAA, SA, ABA, and GA_3_ also decreased. On the contrary, JA content (14.6 ± 0.29 ng g^−1^ FW) and GA_1_ content (1.1 ± 0.03 ng g^−1^ FW) in the roots increased by about four times in comparison to non-treated plants, which were 3.8 ± 0.21 ng g^−1^ FW and 0.3 ± 0.08 ng g^−1^ FW, respectively (Figure 4). Taken together, symbiosis with *P. hingganense* altered phytohormone levels in mung bean plants.

### 3.4. Symbiosis with P. hingganense Enhances Mung Bean Plant Resistance to Diseases Caused by R. solani

**Figure 5:**
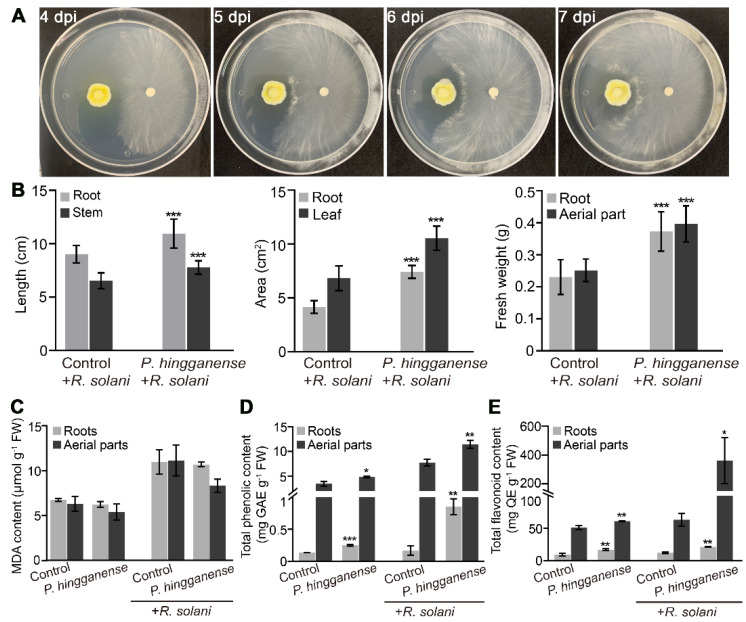
Symbiosis with *P. hingganense* induces resistance to disease caused by *Rhizoctonia solani*. (**A**) Dual culture assay showing moderate antagonistic activity of *P. hingganense* (left) against *R. solani* (right). (**B**) Phenotype comparison of *P. hingganense*-treated and non-treated plants on the 7th day after inoculation of *R. solani*. Values are means ± SD (n = 20). (**C**–**E**) Comparison of MDA content (**C**), total phenolic content (**D**), and total flavonoid content (**E**). Values are means ± SD (n = 3). Asterisks indicate significant differences as evaluated by Student’s *t*-tests: * *p* < 0.05, ** *p* < 0.01, *** *p* < 0.001. MDA, malondialdehyde; GAE, gallic acid equivalent; QE, quercetin equivalent.

Considering the level of defense hormone JA induced in the roots and reduced in the aerial parts upon *P. hingganense* colonization, the response of the mung bean plant to pathogen infection was explored. A dual culture assay showed that *P. hingganense* produced a clear inhibition zone ahead of the *R. solani* colony (Figure 5A), indicating *P. hingganense* exhibited moderated antagonistic activity against *R. solani*. Therefore, *R. solani* was selected as a pathogen agent to assess the role of *P. hingganense* in disease resistance. The wilting index, the hypocotyl lesion index, and the plant lodging index were calculated to show the biocontrol effect of *P. hingganense*. As shown in Table 1, *P. hingganense* decreased the wilting index, the hypocotyl lesion index, and the plant lodging index compared to the control (non-treated plants). Therefore, *P. hingganense* could help mung bean plants resist *R. solani* to some extent. On the other hand, the growth promotion effects of *P. hingganense* also remained, as displayed by greater root and stem length, root and leaf area, and fresh weight (Figure 5B).

In the mung bean plants that showed disease symptoms after *R. solani* infection, lipid peroxidation, which was reflected by MDA content, elevated in both the mycorrhizal plants and non-treated plants compared to plants without *R. solani* infection. The MDA content in the roots of mycorrhizal plants was slightly lower than in non-treated plants, although there was no significant difference between the mycorrhizal plants and non-treated plants (Figure 5C). In contrast, total phenolic and flavonoid contents were significantly enhanced in the mycorrhizal plants (Figure 5D,E). Upon *R. solani* infection, the levels of these compounds were lifted in both mycorrhizal plants and non-treated plants, but were still higher in the mycorrhizal plants (Figure 5D,E).

## 4. Discussion

Plants have benefitted from the mutualistic relationship with fungi since the occurrence of terrestrialization [42]. Both ectomycorrhiza and arbuscular mycorrhiza possess the capacity to boost the growth of plants and impart tolerance to biotic and abiotic stresses [4,5,8]. Given that the primary hosts of ECM fungi are trees, applications of ECM symbionts are mainly focused on forest ecosystems [43]. In this study, the beneficial functions of *P. hingganense* belonging to the novel *Parametarhizum* genus were explored. *P. hingganense* colonized on the root surface and intercellularly developed between the adjacent cells. The establishment of the symbiotic association with *P. hingganense* promoted the growth of mung bean plants and imparted tolerance to the damping off induced by *R. solani*.

The genus *Parametarhizum* was newly discovered and phylogenetically closely related to the genus *Metarhizum*, which has been wildly used as a pesticide [26]. Meanwhile, many *Metarhizum* spp. could colonize the plants and increase growth [36,44,45]. For instance, GFP-tagged *M. anisopliae* was found on the surface and interior of wheat roots [36]. When *M. anisopliae* was used as a seed coating agent, it promoted the growth of wheat shoots and single-spike weight, as well as exhibited biocontrol ability against *Fusarium graminearum* [36]. *M. anisopliae* could also promote the growth of soybeans and alleviate oxidative stress induced by high salinity [46]. Similarly, red fluorescence proteins expressing *M. robertsii* were observed to form a network surrounding the Arabidopsis root and promoted lateral root growth and root hair development [47]. Likewise, *Parametarhizum* was capable of killing three farmland pests: *Monolepta hieroglyphica*, *Callosobruchus chinensis*, and *Rhopalosiphum maidis* [22]. Furthermore, our results showed that *P. hingganense* exhibited moderate anti-fungal activity and formed a symbiotic relationship with mung bean plants. These findings suggested that the capacities of *Parametarhizum* might resemble the members of its sister group. However, the colonization of *Metarhizium* species on plants was demonstrated to be extraradical and inside the root tissue [36]; whether the hyphae penetrate the cell or develop intercellularly was unknown. Given that GFP-tagged *P. hingganense* was located both intercellularly and on the surface of the root, the symbiotic relationship between *P. hingganense* and mung bean plants was more similar to the ectomycorrhizal interaction. In addition, most reported ectomycorrhizal fungi are associated with trees in forests. The forest litter source of *P. hingganense* further supported an ectomycorrhizal symbiont formed by *P. hingganense* and its associated plant.

Enhanced phosphorus uptake is a conserved strategy for mycorrhiza, including ectomycorrhiza and arbuscular mycorrhiza, to promote plant growth [5,7]. However, the performance of mycorrhizal fungi in phosphorus utilization is generally regulated by phosphate bioavailability. The root endophyte *Colletotrichum tofieldiae* only exhibited growth promotion activity on Arabidopsis under limited amounts of Pi [48]. *Glomus intraradices* also only contributed to the phosphorus uptake of *Solidago canadensis* under phosphorus-deficient conditions [49]. Furthermore, fertilization reduced mycorrhizal colonization in six coexisting arbuscular mycorrhizal temperate tree species [50]. During the process of AM fungi colonization, low phosphorus levels in the plants triggers the biosynthesis of strigolactones which will be recognized by AM fungi and lead to the development of AM symbiosis [51]. In contrast, the underlying mechanism of ECM fungi colonization is largely unknown. Research on ECM fungi *L. bicolor*-poplar symbiosis revealed the relevance of *L. bicolor*-produced lipochitooligosaccharides in the establishment of ECM association [52]. In the present study, *P. hingganense* exhibited in vitro phosphate solubilization ability, and the symbiosis with *P. hingganense* led to an increment of Pi content in the host plant, implying that the growth promoting ability of *P. hingganense* should be independent of conditions with phosphorus deficiency. The functional mechanism of *P. hingganense* in phosphorus uptake awaits further investigation.

Despite growth hormones playing crucial roles in mycorrhizal plants, growth promotion is not always accompanied by altered growth hormone levels. *Pochonia chlamydosporia*, another close relative of *P. hingganense*, promotes growth, accelerates floral transition, and enhances yield without changes in GAs and auxin [53]. Here, IAA was absent in *P. hingganense* liquid culture, indicating IAA production was negative for *P. hingganense*. This is further supported by the fact that the IAA level in *P. hingganense*-associated mung bean plants decreased, indicating growth promotion of *P. hingganense* should be independent of the IAA signaling pathway. Nonetheless, it is worth mentioning that GA_1_ in *P. hingganense*-associated roots significantly increased. Although GAs are essential regulators for plant development [10], the roles of GAs in plant-fungi symbiosis were mostly focused on the colonization of mycorrhizal fungi [54]. The complex mechanisms have been well characterized in plant–AM fungi symbiosis [12,51]. GAs acted positively in AM fungi hyphal branching, and negatively in AM fungi entry into the cells [11]. Despite the elevated content of GAs being repeatedly reported in mycorrhizal plants, the growth promotion effect of GAs does not always accompany the reported results. During the development of tobacco–*Glomus intraradices* symbiosis, levels of GA_1_, GA_8_, GA_19_, and GA_20_ were elevated but GA-related growth effect was lacking [55]. Yet, the endogenous levels of GA_1_ and GA_4_ in soybean seedlings co-cultivated with GAs-producing endophytic *Porostereum spadiceum* under salinity stress were enhanced and accompanied by higher shoot growth and increased biomass [56]. Consistently, altered GAs content occurred in the symbiosis of poplar with *L. bicolor*, which led to increased GA_6_ and GA_19_ but decreased GA_4_ [13]. In the present study, given that increased GA_1_ in *P. hingganense*-associated roots was accompanied by obvious growth promotion, GAs could be involved not only in the colonization of *P. hingganense*, but also in contributions to growth promotion.

Enhanced resistance to various stresses is another hallmark of mycorrhizal plants [14]. JA and SA signaling pathways are two key components of mycorrhiza-induced resistance [4]. Here, only JA was induced in *P. hingganense*-associated roots, while SA was repressed. However, resistance to damping off caused by necrotrophic *R. solani* was evident in *P. hingganense*-colonized plants. Despite the influence of JA on AM fungi colonization being controversial, which could be positive, neutral, or negative, the priming function of the JA pathway in the defense system of the host plant is noticeable, especially in the resistance against necrotrophic pathogens [4,12]. The protective effect of ECM fungi colonization was mainly represented by the resistance of host trees to herbivores [4]. Transcriptomic and metabolomic network analysis unraveled the influence of *L. bicolor* association on the JA pathway, which in turn mitigated damage from the poplar leaf beetle *Chrysomela populi* [57]. Therefore, the JA signaling pathway is essential to the defense response of both AM- and ECM-associated plants. In this study, the fact that defense hormones SA and ABA were suppressed in both *P. hingganense*-colonized aerial parts and roots further supported the idea that JA could play an important role in *P. hingganense*-induced local defense response. In addition, *P. hingganense*-induced disease resistance may also result from enriched phenolic and flavonoid content in whole plants, both of which are antioxidants that scavenge excess ROS induced by stresses [58]. This elevation could function in eliminating ROS generated by *P. hingganense* colonization as well, given that no significant difference in MDA content was observed in non-mycorrhizal and mycorrhizal plants. Together, the increment of JA content and antioxidants (phenolic compounds and flavonoids) in *P. hingganense*-colonized plants could strengthen the defense system and lead to tolerance to pathogen infection.

## 5. Conclusions

Here, we presented that *P. hingganense*, a new fungal species of *Parametarhizum* from forest litter, established ectomycorrhizal symbiosis with the crop (mung bean). Promoted growth with elevated Pi occurred in mycorrhizal plants, and enhanced JA and GA_1_ were detected in mycorrhizal roots. Although defense hormones, such as SA and ABA, were repressed, total phenolic and flavonoid content increased due to *P. hingganense* colonization. The improved tolerance of mung bean plants to disease caused by *R. solani* further supported the supposition that induced defense responses were activated upon symbiosis with *P. hingganense*. The underlying mechanism of ECM fungi-induced defense response has not been elucidated yet. Most of the evidence was gathered from the interaction between ECM fungi and host trees. Therefore, our findings demonstrated an alternative strategy for investigation of the beneficial functions of ECM fungi. On the other hand, the beneficial effects of *P. hingganense* on the host plant provide a culturable fungal strain to use as a potential biofertilizer and biocontrol agent.

## Figures and Tables

**Table 1:** Biocontrol efficiency of *Parametarhizum hingganense*.

	Wilting Index	Hypocotyl Lesion Index	Lodging Index
Control	66.25%	71%	66.25%
*P. hingganense*	48.75%	57%	42.5%

## Data Availability

Not applicable.

## Supplementary Materials

The following supporting information can be downloaded at: https://www.mdpi.com/article/10.3390/jof8090934/s1, Figure S1: Colonization of *P. hingganense* on mung bean plants at early stage; Figure S2: Absent of Indole-3-acetic acid (IAA) in *P. hingganense* liquid culture.

## References

[1] Redecker D., Kodner R., Graham L.E. (2000). Glomalean fungi from the Ordovician. Science.

[2] Bonfante P., Genre A. (2010). Mechanisms underlying beneficial plant-fungus interactions in mycorrhizal symbiosis. Nat. Commun..

[3] Brundrett M.C., Tedersoo L. (2018). Evolutionary history of mycorrhizal symbioses and global host plant diversity. New Phytol..

[4] Dreischhoff S., Das I.S., Jakobi M., Kasper K., Polle A. (2020). Local responses and systemic induced resistance mediated by ectomycorrhizal fungi. Front. Plant Sci..

[5] Smith S.E., Smith F.A. (2011). Roles of arbuscular mycorrhizas in plant nutrition and growth: New paradigms from cellular to ecosystem scales. Annu. Rev. Plant Biol..

[6] Poirier Y., Jaskolowski A., Clua J. (2022). Phosphate acquisition and metabolism in plants. Curr. Biol..

[7] Landeweert R., Hoffland E., Finlay R.D., Kuyper T.W., van Breemen N. (2001). Linking plants to rocks: Ectomycorrhizal fungi mobilize nutrients from minerals. Trends Ecol. Evol..

[8] Becquer A., Trap J., Irshad U., Ali M.A., Claude P. (2014). From soil to plant, the journey of P through trophic relationships and ectomycorrhizal association. Front. Plant Sci..

[9] Yu Z., Zhang F., Friml J., Ding Z. (2022). Auxin signaling: Research advances over the past 30 years. J. Integr. Plant Biol..

[10] Silverstone A.L., Sun T. (2000). Gibberellins and the Green Revolution. Trends Plant Sci..

[11] Takeda N., Handa Y., Tsuzuki S., Kojima M., Sakakibara H., Kawaguchi M. (2015). Gibberellins interfere with symbiosis signaling and gene expression and alter colonization by arbuscular mycorrhizal fungi in Lotus japonicus. Plant Physiol..

[12] Liao D., Wang S., Cui M., Liu J., Chen A., Xu G. (2018). Phytohormones regulate the development of arbuscular mycorrhizal symbiosis. Int. J. Mol. Sci..

[13] Basso V., Kohler A., Miyauchi S., Singan V., Guinet F., Simura J., Novak O., Barry K.W., Amirebrahimi M., Block J. (2020). An ectomycorrhizal fungus alters sensitivity to jasmonate, salicylate, gibberellin, and ethylene in host roots. Plant Cell Environ..

[14] Jung S.C., Martinez-Medina A., Lopez-Raez J.A., Pozo M.J. (2012). Mycorrhiza-induced resistance and priming of plant defenses. J. Chem. Ecol..

[15] Zhang Y.C., Zou Y.N., Liu L.P., Wu Q.S. (2019). Common mycorrhizal networks activate salicylic acid defense responses of trifoliate orange (*Poncirus trifoliata*). J. Integr. Plant Biol..

[16] Sandoval R.F.C., Cumagun C.J.R. (2019). Phenotypic and molecular analyses of *Rhizoctonia* spp. associated with rice and other hosts. Microorganisms.

[17] Anderson N.A. (1982). The genetics and pathology of *Rhizoctonia solani*. Annu. Rev. Phytopathol..

[18] Tsror L. (2010). Biology, Epidemiology and management of *Rhizoctonia solani* on potato. J. Phytopathol..

[19] Kataria H.R., Grover R.K. (1978). Comparison of fungicides for the control of *Rhizoctonia solani* causing datnping-off of mung bean (*Phaseohis aureus*). Ann. Appl. Biol..

[20] Dubey S.C., Bhavani R., Singh B. (2011). Integration of soil application and seed treatment formulations of *Trichoderma* species for management of wet root rot of mungbean caused by *Rhizoctonia solani*. Pest Manag. Sci..

[21] Carling D.E., Baird R.E., Gitaitis R.D., Brainard K.A., Kuninaga S. (2002). Characterization of AG-13, a newly reported anastomosis group of *Rhizoctonia solani*. Phytopathology.

[22] Gao S., Meng W., Zhang L., Yue Q., Zheng X., Xu L. (2021). *Parametarhizium* (*Clavicipitaceae*) gen. nov. with two new species as a potential biocontrol agent isolated from forest litters in Northeast China. Front. Microbiol..

[23] Sung G.H., Sung J.M., Hywel-Jones N.L., Spatafora J.W. (2007). A multi-gene phylogeny of Clavicipitaceae (Ascomycota, Fungi): Identification of localized incongruence using a combinational bootstrap approach. Mol. Phylogenet. Evol..

[24] Schardl C.L., Young C.A., Hesse U., Amyotte S.G., Andreeva K., Calie P.J., Fleetwood D.J., Haws D.C., Moore N., Oeser B. (2013). Plant-symbiotic fungi as chemical engineers: Multi-genome analysis of the clavicipitaceae reveals dynamics of alkaloid loci. PLoS Genet..

[25] Mongkolsamrit S., Khonsanit A., Thanakitpipattana D., Tasanathai K., Noisripoom W., Lamlertthon S., Himaman W., Houbraken J., Samson R.A., Luangsa-ard J. (2020). Revisiting *Metarhizium* and the description of new species from Thailand. Stud. Mycol..

[26] St Leger R.J., Wang J.B. (2020). *Metarhizium*: Jack of all trades, master of many. Open Biol..

[27] Wang Y., Wang L., Suo M., Qiu Z., Wu H., Zhao M., Yang H. (2022). Regulating root fungal community using *Mortierella alpina* for *Fusarium oxysporum* resistance in *Panax ginseng*. Front. Microbiol..

[28] Schneider C.A., Rasband W.S., Eliceiri K.W. (2012). NIH Image to ImageJ: 25 years of image analysis. Nat. Methods.

[29] Doilom M., Guo J.W., Phookamsak R., Mortimer P.E., Karunarathna S.C., Dong W., Liao C.F., Yan K., Pem D., Suwannarach N. (2020). Screening of phosphate-solubilizing fungi from air and soil in Yunnan, China: Four novel species in *Aspergillus*, *Gongronella*, *Penicillium*, and *Talaromyces*. Front. Microbiol..

[30] Nautiyal C.S. (1999). An efficient microbiological growth medium for screening phosphate solubilizing microorganisms. FEMS Microbiol. Lett..

[31] Chiou T.J., Aung K., Lin S.I., Wu C.C., Chiang S.F., Su C.L. (2006). Regulation of phosphate homeostasis by MicroRNA in *Arabidopsis*. Plant Cell.

[32] Bose A., Shah D., Keharia H. (2013). Production of indole-3-acetic-acid (IAA) by the white rot fungus *Pleurotus ostreatus* under submerged condition of Jatropha seedcake. Mycology.

[33] Zhang A., Lu P., Dahl-Roshak A.M., Paress P.S., Kennedy S., Tkacz J.S., An Z. (2003). Efficient disruption of a polyketide synthase gene (*pks1*) required for melanin synthesis through *Agrobacterium*-mediated transformation of *Glarea lozoyensis*. Mol. Genet. Genom..

[34] Pan X., Welti R., Wang X. (2010). Quantitative analysis of major plant hormones in crude plant extracts by high-performance liquid chromatography-mass spectrometry. Nat. Protoc..

[35] Meng W., Xu L., Du Z.Y., Wang F., Zhang R., Song X., Lam S.M., Shui G., Li Y., Chye M.L. (2020). RICE ACYL-COA-BINDING PROTEIN6 affects acyl-coa homeostasis and growth in rice. Rice.

[36] Hao Q.Y., Albaghdady D.M.D., Xiao Y.N., Xiao X.Q., Mo C.M., Tian T., Wang G.F. (2021). Endophytic *Metarhizium anisopliae* is a potential biocontrol agent against wheat Fusarium head blight caused by *Fusarium graminearum*. J. Plant Pathol..

[37] Lisker N., Katan J., Henis Y. (1980). Lesion formation on bean seedling hypocotyls by *Rhizoctonia solani* as affected by size and nutrition of propagules. Ann. Bot..

[38] Wang G., Zhou Q., He M., Zhong X., Tang G. (2020). Wilting index and root morphological characteristics used as drought-tolerance variety selection at the seedling stage in soybean (*Glycine max* L.). Plant Growth Regul..

[39] Guo Z.H., Pogancev G., Meng W., Du Z.Y., Liao P., Zhang R., Chye M.L. (2021). The overexpression of rice ACYL-COA-BINDING PROTEIN4 improves salinity tolerance in transgenic rice. Environ. Exp. Bot..

[40] Martinez-Arias C., Sobrino-Plata J., Ormeno-Moncalvillo S., Gil L., Rodriguez-Calcerrada J., Martin J.A. (2021). Endophyte inoculation enhances *Ulmus minor* resistance to Dutch elm disease. Fungal Ecol..

[41] Kurepin L.V., Zaman M., Pharis R.P. (2014). Phytohormonal basis for the plant growth promoting action of naturally occurring biostimulators. J. Sci. Food Agric..

[42] Rich M.K., Vigneron N., Libourel C., Keller J., Xue L., Hajheidari M., Radhakrishnan G.V., Le Ru A., Diop S.I., Potente G. (2021). Lipid exchanges drove the evolution of mutualism during plant terrestrialization. Science.

[43] Policelli N., Horton T.R., Hudon A.T., Patterson T.R., Bhatnagar J.M. (2020). Back to Roots: The role of ectomycorrhizal fungi in boreal and temperate forest restoration. Front. Glob. Chang..

[44] Barelli L., Moreira C.C., Bidochka M.J. (2018). Initial stages of endophytic colonization by *Metarhizium* involves rhizoplane colonization. Microbiology.

[45] Jiang X., Fang W., Tong J., Liu S., Wu H., Shi J. (2022). *Metarhizium robertsii* as a promising microbial agent for rice in situ cadmium reduction and plant growth promotion. Chemosphere.

[46] Khan A.L., Hamayun M., Khan S.A., Kang S.M., Shinwari Z.K., Kamran M., Ur Rehman S., Kim J.G., Lee I.J. (2012). Pure culture of *Metarhizium anisopliae* LHL07 reprograms soybean to higher growth and mitigates salt stress. World J. Microbiol. Biotechnol..

[47] Liao X., Lovett B., Fang W., St Leger R.J. (2017). *Metarhizium robertsii* produces indole-3-acetic acid, which promotes root growth in *Arabidopsis* and enhances virulence to insects. Microbiology.

[48] Hiruma K., Gerlach N., Sacristan S., Nakano R.T., Hacquard S., Kracher B., Neumann U., Ramirez D., Bucher M., O’Connell R.J. (2016). Root endophyte *Colletotrichum tofieldiae* confers plant fitness benefits that are phosphate status dependent. Cell.

[49] Qi S., Wang J., Wan L., Dai Z., da Silva Matos D.M., Du D., Egan S., Bonser S.P., Thomas T., Moles A.T. (2022). Arbuscular mycorrhizal fungi contribute to phosphorous uptake and allocation strategies of *Solidago canadensis* in a phosphorous-deficient environment. Front. Plant Sci..

[50] Eissenstat D.M., Kucharski J.M., Zadworny M., Adams T.S., Koide R.T. (2015). Linking root traits to nutrient foraging in arbuscular mycorrhizal trees in a temperate forest. New Phytol..

[51] Ho-Plagaro T., Garcia-Garrido J.M. (2022). Molecular regulation of arbuscular mycorrhizal symbiosis. Int. J. Mol. Sci..

[52] Cope K.R., Bascaules A., Irving T.B., Venkateshwaran M., Maeda J., Garcia K., Rush T.A., Ma C., Labbe J., Jawdy S. (2019). The ectomycorrhizal fungus *Laccaria bicolor* produces lipochitooligosaccharides and uses the common symbiosis pathway to colonize populus roots. Plant Cell.

[53] Zavala-Gonzalez E.A., Rodriguez-Cazorla E., Escudero N., Aranda-Martinez A., Martinez-Laborda A., Ramirez-Lepe M., Vera A., Lopez-Llorca L.V. (2017). *Arabidopsis thaliana* root colonization by the nematophagous fungus *Pochonia chlamydosporia* is modulated by jasmonate signaling and leads to accelerated flowering and improved yield. New Phytol..

[54] Foo E., Ross J.J., Jones W.T., Reid J.B. (2013). Plant hormones in arbuscular mycorrhizal symbioses: An emerging role for gibberellins. Ann. Bot..

[55] Shaul-Keinan O., Gadkar V., Ginzberg I., Grunzweig J.M., Chet I., Elad Y., Wininger S., Belausov E., Eshed Y., Atzmon N. (2002). Hormone concentrations in tobacco roots change during arbuscular mycorrhizal colonization with *Glomus intraradices*. New Phytol..

[56] Hamayun M., Hussain A., Khan S.A., Kim H.Y., Khan A.L., Waqas M., Irshad M., Iqbal A., Rehman G., Jan S. (2017). Gibberellins producing endophytic fungus *Porostereum spadiceum* AGH786 rescues growth of salt affected soybean. Front. Microbiol..

[57] Kaling M., Schmidt A., Moritz F., Rosenkranz M., Witting M., Kasper K., Janz D., Schmitt-Kopplin P., Schnitzler J.P., Polle A. (2018). Mycorrhiza-triggered transcriptomic and metabolomic networks impinge on herbivore fitness. Plant Physiol..

[58] Foyer C.H., Noctor G. (2005). Redox homeostasis and antioxidant signaling: A metabolic interface between stress perception and physiological responses. Plant Cell.

