# Supplementary material for: Parametarhizium hingganense, a Novel Ectomycorrhizal Fungal Species, Promotes the Growth of Mung Beans and Enhances Resistance to Disease Induced by Rhizoctonia solani
Source: J Fungi (Basel). 2022 Sep 2;8(9):934. doi: 10.3390/jof8090934 (PMC9504979; doi:10.3390/jof8090934)
Supplement: Supplementary file 1 [file jof-08-00934-s001.zip › Supplementary Materials.pdf]

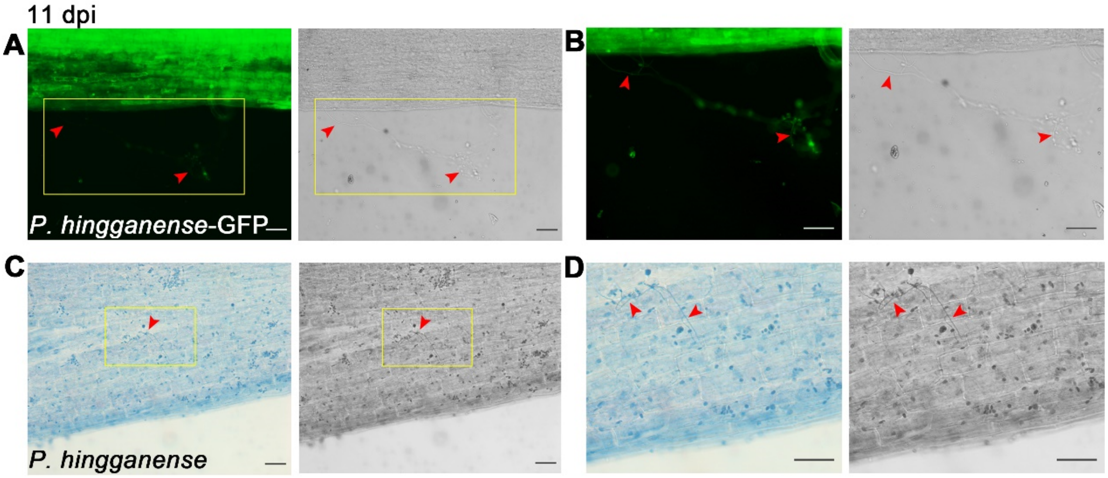

Supplementary Figure S1. Colonization of *P. hingganense* on mung bean plants at early stage. (**A**) Fluorescence microscope images showing the hyphae of GFP–tagged *P. hingganense* on the roots at 11 dpi. (**B**) Enlargement of the rectangle in (A). (**C**) Light microscope images of trypan blue staining of the hyphae of *P. hingganense* at 11 dpi. (**D**) Enlargement of the rectangle in (C). Bars, 50 μm. Yellow rectangles indicate root areas that are enlarged. Red arrow heads, hyphae.

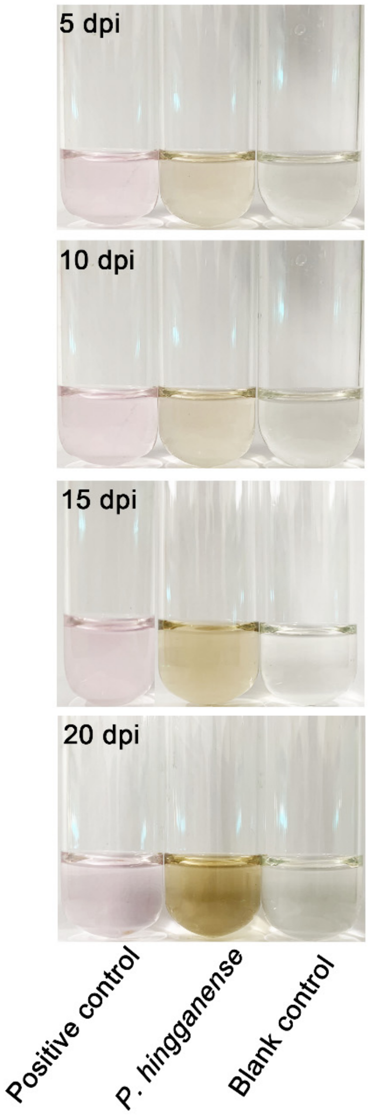

Supplementary Figure S2. Absent of Indole–3–acetic acid (IAA) in *P. hingganense* liquid culture. *P. hingganense* was inoculated and grown in Potato Dextrose (PD) liquid medium supplemented with 0.1% (w/v) L–tryptophan and sampled at 5, 10, 15, and 20 dpi. Positive control, pink color shows the reaction of IAA with Salkowski reagent. Blank control, PD liquid medium without L–tryptophan.
